# Secondary analysis of potential associations between oral health and infection-related parameters in patients with severe heart failure—results of a German cohort

**DOI:** 10.1186/s12872-023-03612-1

**Published:** 2023-11-21

**Authors:** Gerhard Schmalz, Alina Hennecke, Rainer Haak, Tanja Kottmann, Jens Garbade, Christian Binner, Dirk Ziebolz

**Affiliations:** 1 Department of Cariology, Endodontology and Periodontology, University Leipzig, Liebigstr. 12, D 04103 Leipzig, Germany; 2 CRO Dr. med. Kottmann GmbH & Co. KG, Hamm, Germany; 3 Department of Cardiac Surgery, Klinikum Links der Weser, 28277 Bremen, Germany; 4 University Department of Cardiac Surgery, Heart Center Leipzig, Leipzig, Germany

**Keywords:** Heart Failure, Periodontitis, Periodontal medicine, Infection, Oral health

## Abstract

**Objectives:**

Aim of this retrospective cohort study was to evaluate whether oral health parameters would be associated with infection-related parameters and overall survival of patients with severe heart failure (HF).

**Methods:**

Patients with severe HF, heart transplantation (HTx) and left-ventricular assist device (LVAD), which underwent a full oral examination between 2017 and 2018 were included. Infection-related and survival data were assessed from the patient´s medical records. The oral examination included: remaining teeth, caries and periodontal condition, including periodontal probing depth (PPD), clinical attachment loss (CAL), bleeding on probing (BOP), and diagnosis (staging/grading). In addition, the periodontal inflamed surface area (PISA) was determined. Statistical analysis included Chi-square, Fisher´s exact and Mann-Whitney-U test, as well as a logistic regression, considering age, gender, body-mass-index (BMI), diabetes and several oral health parameters with regard to overall survival and infections at heart/driveline.

**Results:**

329 patients (HTx: 34%, LVAD: 38.9%, HF: 27.1%), were included. Patients had on average 18.96 ± 8.90 remaining teeth, whereby the majority had a periodontitis stage III or IV (88.7%) and a grade B (80.5%). Higher BOP was associated with infections at heart/driveline (p = 0.04) and outside the heart (p < 0.01) during follow-up. Increased PISA was significantly associated with bacterial infections outside the heart (p < 0.01) and sepsis (p = 0.02). Only BMI of 25 or higher correlated with an increased risk of infections at heart/driveline in regression analysis (OR 3.063, CI_95_ 1.158–8.101, p = 0.02), while no associations between oral health parameters and infections at heart/driveline or overall survival were confirmed.

**Conclusions:**

In patients with severe HF, periodontal inflammation might be associated with infection-related parameters. Improved dental care, especially including periodontal therapy and maintenance might be favourable to support prevention of infections in patients with severe HF.

**Supplementary Information:**

The online version contains supplementary material available at 10.1186/s12872-023-03612-1.

## Introduction

The link between periodontitis and heart diseases, especially coronary heart diseases has been comprehensively described; thereby both, periodontal diseases as well as its long-term consequences, i.e. tooth loss are related to the risk of heart diseases [1–3]. Thereby, a consensus report between European Federation of Periodontology (EFP) and World Heart Federation (WHF) was organized to discuss on periodontitis and cardiovascular diseases, and underlined the necessity for periodontal therapy and maintenance in individuals suffering from heart diseases [4].

In contrast to those recommendations, patients with severe heart diseases, particularly severe heart failure (HF) show an enormous prevalence of oral and periodontal diseases. Several recent studies showed periodontitis severity to be high and oral health behaviour to be reduced in patients with HF, which has also been found in case of treatment with heart transplantation (HTx) and left ventricular assist device (LVAD) [5–7]. A prospective study in a German center showed that there is a lack in periodontal care of patients with HF, because although patients were referred to their dentists for treatment, their periodontal treatment need remained high [8]. This could be of high clinical relevance, because periodontal treatment and the respective therapy response could predict cardiovascular events [9]. A recent meta-analysis concluded that periodontitis is associated with mortality due to cardiovascular diseases [3]. Similarly, all types of edentulism were found to be associated with mortality risk (all-cause mortality) [3]. Those conclusions are supported by recent large-scaled clinical studies; one out of them had an observational period of 17 years and was able to show that periodontitis leads to increased risk for future events of ischemic heart diseases and death [10]. The causal underlying mechanisms remain unclear, whereby a large recent investigation could not confirm a role of systemic inflammation as causal explanation for periodontitis and tooth loss as risk predictors of mortality [11].

Against this background, the role of periodontal burden and tooth loss as potential influential factors on the occurrence of infectious complications could be of interest. One recent retrospective study in patients with LVAD did not reveal a role of periodontal parameters for infection and/or microbiological colonization of the driveline [12]. However, a potentially relevant role of periodontal disease and related pathogens in the occurrence of cardiac infections, e.g. infective endocarditis appears probable [13, 14]. Especially for patients with severe HF, after HTx and with LVAD, clinical data on the potential role of oral and especially periodontal conditions for cardiological outcome are still rare. Therefore, this current study aimed to evaluate whether oral health parameters would be associated with infection-related parameters, particularly cardiac and/or systemic infections as well as overall survival. For this purpose, a cohort of patients with severe HF, HTx and LVAD was investigated retrospectively in 2021, after an initial comprehensive oral examination between 2017 and 2018 [5, 6]. It was hypothesized that severity of periodontitis and tooth loss would be associated with the occurrence of infections as well as survival of the patients.

## Methods

This retrospective cohort study based on a cooperation between the Department for Cardiac Surgery at the Heart Center Leipzig and the Department of Cariology, Endodontology and Periodontology, University of Leipzig and included a described patients cohort from a previous clinical observational study [5, 6]. The study was performed according to the Declaration of Helsinki. The study has been reviewed and approved by the ethics committee of the Medical Faculty of University of Leipzig (No: 414/16-ek).

### Patients

For this retrospective cohort study, patients, who underwent an oral examination between May 2017 and December 2018 during their routine follow-up appointment at the Department for Cardiac Surgery at the Heart Center Leipzig were included. The cohort comprised of patients regularly attending the Heart Center because of heart failure without circulatory support (HF, in previous studies given as “Heart insufficiency”), heart transplantation (HTx) or left ventricular assist device (LVAD). No sample size calculation was performed, while the size of the cohort was determined by the patients, which were initially investigated. At the time point before dental examination, all participants were informed about the study and provided written informed consent for participation. This informed consent included a subsequent assessment and analysis of follow-up data, which were assessed for all suitable patients in 2021. Specific inclusion criteria for the current study were therefore: full oral examination in 2017/2018, written informed consent for subsequent analysis of clinical outcome data and follow-up in Heart Center Leipzig after oral examination. The exclusion criteria were according to the previously performed oral examination [5, 6]:

- worse general health status making oral examination impossible, e.g. critically ill or acute life-threatening condition
- autoimmune diseases (e.g. rheumatoid arthritis)
- infectious diseases (hepatitis A, B, C, tuberculosis, HIV),
- pregnancy

### Collection of clinical cardiological outcome data

The outcome data were assessed from the medical records of the patients. One experienced researcher extracted the patient outcome parameters from the records of each included patient case. Thereby, the following parameters were evaluated:

- Number of in-patient stays in 2 years after dental examination
- Regular ambulant maintenance in Heart Center (regular means at least 2 maintenance visits annually)
- Infections at heart or driveline (LVAD patients) during the observational period (only bacterial infections were counted)
- Bacterial infections outside heart/driveline during the observational period
- Sepsis during the study period
- Viral infection during the study period
- Survival
- Reason for death in case of death during the study period (cardiac, infection or other reasons)

Additionally, demographic data, including age, gender, smoking habits, co-morbidities, underlying heart diseases and several medications as well as body-mass-index (BMI) were extracted from the patient records.

### Oral examination

The oral examinations have been described in detail previously [5, 6]. All investigations were executed by three experienced and calibrated (kappa > 0.8) dentists under same conditions at the Department for Cardiac Surgery at Heart Center Leipzig. If necessary (HTx and LVAD), patients received 2 g Amoxicillin (or 600 mg Clindamycine in case of an allergy against penicillin) as an antibiotic prophylaxis an hour prior to examination [15].

In brief, oral examination included dental and periodontal findings. A visual inspection of the oral cavity using mirror and probe was carried out to record the number of remaining teeth, remaining molars/premolars and front teeth and the presence of caries with a cavitation of the tooth surface (D-T); in case of at least one carious lesion, dental treatment need was stated. The periodontal examination of each dentate patient, included periodontal probing depth (PPD), clinical attachment loss (CAL) and bleeding on probing (BOP) at six measurement points per tooth using a millimeter scaled periodontal probe^1^. Based on the current classification of periodontal diseases, the diagnosis of periodontitis, i.e. stage (stage I—IV) and grade (grade: A-C) were determined [16]. Additionally, the periodontal inflamed surface area (PISA) was assessed according to Nesse et al. [17].

### Statistical analysis

The statistical analysis was performed with SPSS for Windows, version 24.0^2^. Prior to analysis, Kolmogorov-Smirnov-test was applied to assess whether parameters were normal distributed. Thereby, none of the investigated parameters was normal distributed (p < 0.05). Categorical and nominal data were compared using chi-square or Fishers exact test, respectively. Two independent, non-normal distributed samples were compared with Mann-Whitney-U test. The significance level has been set at p < 0.05. The multivariate analysis included a binary logistic regression analysis, whereby either survival or infections at heart or driveline were the dependent variable.

## Results

### Patients

**Fig. 1:**
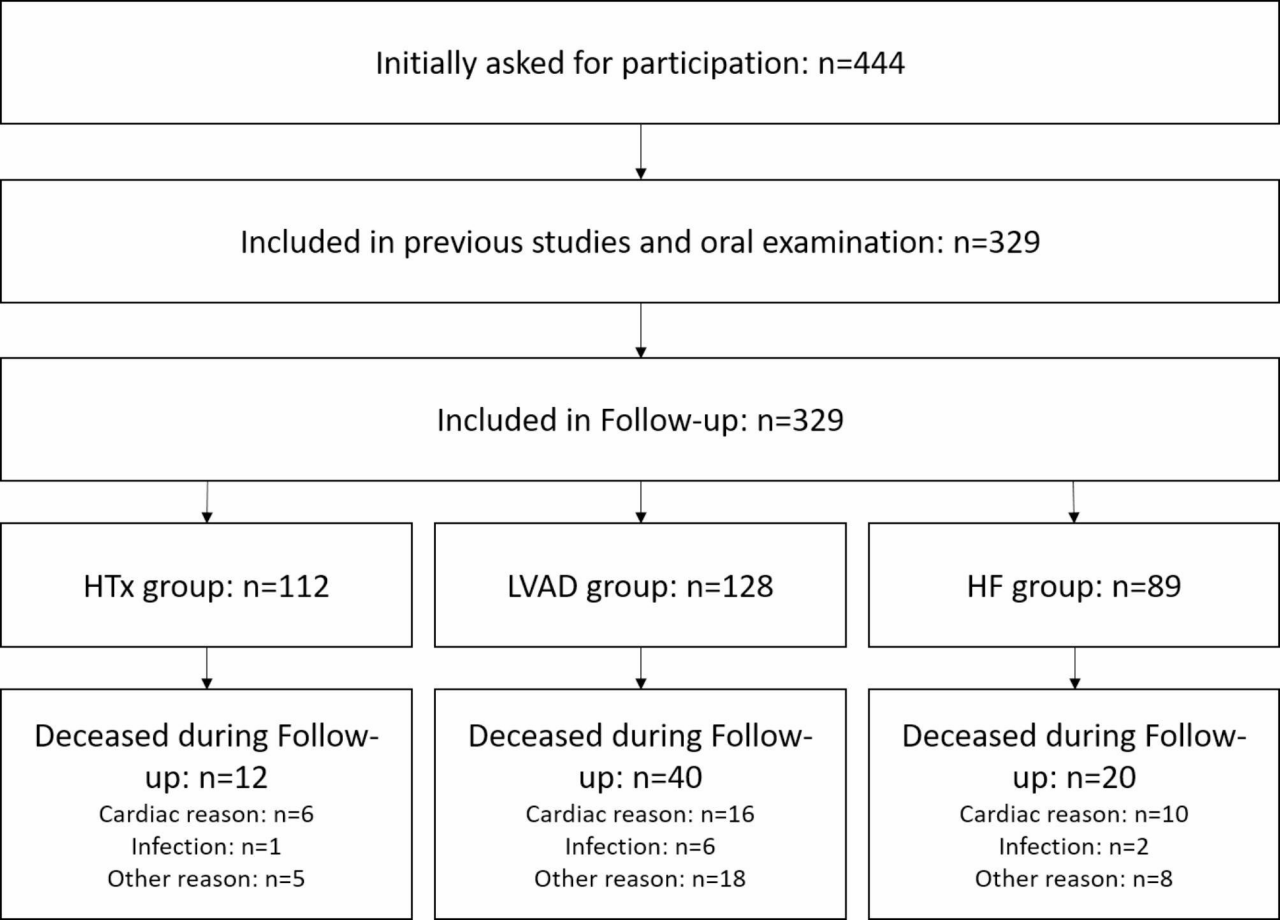
flow-chart of the patients in this secondary analysis,

In total, 329 patients (HTx: 34%, LVAD: 38.9%, HF: 27.1%), with a mean age of 57.3 ± 11.55 years and an amount of 84.5% of male gender were included in this retrospective cohort study. The patient characteristics are displayed in Table 1 and a flow-chart is given as Fig. 1.

**Table 1:** Patient characteristics and cardiological outcome data

			Patients with HF (n = 329)
**Gender (male in % [n])**			84.5% [278]
**Age in years (mv ± SD [median; range])**			57.3 ± 11.55 [60; 22–85]
**Smoking habits ** **% [n]**	smoker		12.2% [40]
	non-smoker		87.8% [289]
**Co-morbidities % [n]**	hypertension		67.5% [222]
	diabetes mellitus		39.2% [129]
	renal insufficiency		62.6% [206]
	osteoporosis		3% [10]
**Underlying cardiac disease**	ICM		36.5% [120]
	DCM		55.6% [183]
	Infarction		20.4% [67]
	Heart valve insufficiency		34.7% [114]
	Auricular/ventricular fibrillation		28% [92]
	Myocarditis/Endocarditis		8.2% [27]
	Coronary heart disease		32.8% [108]
**Ejection fraction in % (mv ± SD)**			32.61 ± 16.80
**Body-Mass-Index BMI (mv ± SD)**			28.22 ± 4.78
**BMI ≥ 25**			74.2% [244]
**Disease/** **Therapy situation**	HTx		34% [112]
	LVAD		38.9% [128]
	HF without HTx/LVAD		27.1% [89]
**Medication**	Anticoagulants		59.3% [195]
	Immunosuppressive		26.7% [88]
	Insulin		19.1% [63]
	Oral antidiabetic drugs		16.4% [54]
	Bisphosphonates		13.1% [43]
**Number of in-patients stays in 2 years after dental examination**			2.05 ± 2.51
**Regular ambulant maintenance in LHC**			51.4% [169]
**Infection at heart/driveline**			15.5% [51]
**Bacterial infection outside heart/driveline**			12.8% [42]
**Sepsis**			10.9% [36]
**Viral infection**			6.1% [20]
**Overall survival**			77.5% [255]
**Reason for death**		**Cardiac**	44.4% [32/72]
		**Infection**	12.5% [9/72]
		**Others**	43.1% [31/72]

ICM: ischemic cardiomyopathy, DCM: dilatative cardiomyopathy, HTX: heart transplantation, LVAD: left ventricular assist device, HF: heart failure, LHC: Leipzig Heart Center, SD: standard deviation, mv: mean value

### Overall cardiological outcomes

The cardiological outcomes are shown in Table 1. The prevalence of infection at heart/driveline was 15.5% and outside heart/driveline 12.8%. During the study period, 22.5% died, mainly due to cardiological reasons (44.4%).

## Oral health findings

Most individuals were dentate (93.6%), with on average 18.96 ± 8.90 remaining teeth. The majority had a periodontitis stage III or IV (89.1%) and a grade B (80.5%, Table 2).

**Table 2:** Oral and periodontal findings. Values are either given as means and standard deviation or as percentage and number of patients

		Patients with HF (n = 329)
Dental findings		
**Edentulous**		6.4% [21]
**Remaining teeth (mv ± SD)**		18.96 ± 8.90
**Remaining molars/premolars** **(mv ± SD)**		9.56 ± 5.4
**Remaining front teeth (mv ± SD)**		9.38 ± 3.96
**D-T**		0.37 ± 1.15
**Dental treatment need**		16.7% [55]
**Periodontal findings**		
**PPD mean in mm (mv ± SD)**		2.96 ± 0.70
**Number of teeth PPD ≥ 5 mm (mv ± SD)**		5.96 ± 6.04
**CAL mean in mm (mv ± SD)**		2.94 ± 1.27
**Number of teeth CAL ≥ 5 mm (mv ± SD)**		4.99 ± 5.50
**BOP % (mv ± SD)**		19.23 ± 18.57
**PISA in mm² (mv ± SD)**		271.03 ± 311.06
**Gingival overgrowth % [n]**		4.6% [15]
**Periodontitis stage % [n]**	**I**	0.6% [2/301]
	**II**	10.3% [31/301]
	**III**	41.9% [126/301]
	**IV**	47.2% [142/301]
**Periodontitis grade % [n]**	**B**	80.5% [242/301]
	**C**	19.5% [59/301]

CAL: clinical attachment loss, PPD: periodontal probing depth, BOP: bleeding on probing, PISA: periodontal inflamed surface area, D-T: number of decayed teeth

### Associations between dental health and infection-related parameters and survival

Dental health conditions, i.e. the number of remaining teeth, dental treatment need or the caries prevalence (D-T) were not associated to infection-related cardiological outcomes as well as overall survival of the included patients (p > 0.05, Table 3).

**Table 3:** Associations between dental conditions and remaining teeth with infectious complications as well as survival. The table compares the prevalence between different sub-groups regarding dental conditions; those sub-groups were composed either by median/stratification of the results in the overall cohort or dichotomous in case of yes/no decisions

		Number of patients	Infections at heart/driveline	Bacterial infections outside heart	Sepsis	Overall survival
**Number of remaining teeth**	≤ 13	82	19.5%	12.2%	15.9%	74.4%
	14–23	85	11.8%	14.1%	9.4%	70.2%
	24–26	81	16%	17.3%	11.1%	81.3%
	27+	73	15.1%	6.8%	6.8%	84.9%
	*p*-value	-	0.57	0.27	0.32	0.12
**Remaining molars/** **premolars**	≤ 5	91	19.8%	12.1%	16.5%	74.7%
	6–11	72	12.5%	15.3%	8.3%	71.8%
	12–14	80	13.8%	17.5%	11.3%	77.5%
	15+	78	15.4%	6.4%	6.4%	85.7%
	*p*-value	-	0.58	0.18	0.17	0.20
**Remaining front teeth**	≤ 8	89	19.1%	12.4%	14.6%	73%
	9–12	232	14.2%	12.9%	9.5%	79.1%
	*p*-value	-	0.30	0.99	0.23	0.30
**Dental treatment need**	Yes	267	14.8%	13%	13%	75.9%
	No	54	15.7%	12.7%	10.5%	77.7%
	*p*-value	-	0.99	0.99	0.63	0.86
**D-T**	≤ 0	274	15.7%	12.7%	10.5%	77.7%
	1+	55	14.8%	13%	13%	75.9%
	*p*-value	-	0.99	0.99	0.63	0.86

D-T: number of decayed teeth, significant values are highlighted in bold (chi-square test, p < 0.05)

### Associations between periodontal findings and infection-related parameters and survival

A higher BOP was associated with infections at heart/driveline (p = 0.04) and bacterial infections outside the heart (p < 0.01). Furthermore, an increased PISA was significantly associated with bacterial infections outside the heart (p < 0.01) and sepsis (p = 0.02; Table 4). No associations were found between periodontal parameters and overall survival (p > 0.05).

**Table 4:** Associations between periodontal findings and infectious complications as well as survival. The table compares the prevalence between different sub-groups regarding dental conditions; those sub-groups were composed either by median/stratification of the results in the overall cohort or dichotomous in case of yes/no decisions

		Number of patients	Infections at heart/driveline	Bacterial infections outside heart	Sepsis	Overall survival
**PPD mean**	≤ 2.5	74	17.6%	10.8%	12.2%	75.3%
	2.51–2.8	75	16%	20%	13.3%	81.3%
	2.81–3.35	76	15.8%	10.5%	11.8%	82.9%
	3.36+	74	13.5%	9.5%	6.8%	74.0%
	*p*-value	-	0.93	0.18	0.59	0.47
**PPD > 5 mm**	≤ 1	87	14.9%	11.5%	9.2%	76.7%
	2–4	76	17.1%	7.9%	10.5%	78.9%
	5–9	66	16.7%	13.6%	13.6%	87.9%
	10+	72	13.9%	19.4%	11.1%	70.4%
	*p*-value	-	0.95	0.20	0.86	0.10
**BOP**	≤ 7	86	14%	2.3%	5.8%	73.3%
	8–14	67	6%	13.4%	9%	80.6%
	15–26	77	19.5%	20.8%	13%	75.3%
	27+	71	22.5%	16.9%	16.9%	85.5%
	*p*-value	**-**	**0.04**	**< 0.01**	0.14	0.26
**Periodontitis stage**	1	2	0%	0%	0%	100%
	2	31	32.3%	6.5%	9.7%	74.2%
	3	126	12.7%	15.1%	9.5%	84.8%
	4	142	14.8%	12.7%	12.7%	73%
	*p*-value	-	0.05	0.58	0.80	0.10
**Periodontitis grade**	B	242	14.9%	13.6%	10.3%	80.4%
	C	58	19%	10.3%	13.8%	69%
	*p*-value	-	0.68	0.74	0.71	0.14
**PISA**	≤ 64.84	76	15.8%	7.9%	10.5%	75%
	64.85-174.59	74	13.5%	2.7%	2.7%	75.7%
	174.6-379.12	77	13%	18.2%	18.2%	76.3%
	379.13+	75	20%	22.7%	12%	86.5%
	*p*-value	-	0.63	**< 0.01**	**0.02**	0.27

PPD: periodontal probing depth, BOP: bleeding on probing, PISA: periodontal inflamed surface area, significant values are highlighted in bold (chi-square test, p < 0.05)

### Logistic regression analysis

A higher age of the included individuals correlated significantly with worse survival in the current study (OR 0.970, CI_95_ 0.944–0.996, p = 0.03; Table 5). With infections at heart/driveline as the dependent variable, it could be revealed that a BMI of 25 or higher correlated with a three-fold increased risk of infections (OR 3.063, CI_95_ 1.158–8.101, p = 0.02; Table 6).

**Table 5:** Logistic regression analysis of overall survival (independent of cardiological, infectious or other cause) as dependent variable for different potential influential factors

		B	*p*-value	OR	95% Confidence interval for OR	
					lower	upper
Step 1^a^	Age	-0.023	0.12	0.977	0.949	1,006
	Gender(m)	0.013	0.98	10,013	0.428	2,401
	BMI	-0.008	0.81	0.992	0.928	1,060
	Diabetes	-0.226	0.49	0.798	0.420	1,514
	Number of remaining teeth	0.004	0.85	1.004	0.966	1,043
	PPD	-0.039	0.16	0.962	0.911	1,016
	PISA	0.001	**0.04**	1.001	1.000	1,003
	Constant	2.551	0.08	12.821		
Step 7^a^	Age	-0.031	**0.03**	0.970	0.944	0.996
	PISA	0.001	0.08	10.001	1.000	1.002
	Constant	2.815	< 0.01	160.687		

BMI: body mass index, PPD: periodontal probing depth, PISA: periodontal inflamed surface area, significant values are highlighted in bold (p < 0.05)

**Table 6:** Logistic regression analysis of infections at heart/driveline as dependent variable for different potential influential factors

		B	*p*-value	OR	95% Confidence interval for OR	
					lower	upper
Step 1^a^	Age	-0.006	0.70	0.994	0.964	1.025
	Gender(m)	1.037	0.10	2.822	0.811	9.813
	BMI (≥ 25)	1.145	**0.03**	3.144	1.146	8.627
	Diabetes	0.181	0.63	1.199	0.578	2.488
	Number of remaining teeth	-0.011	0.62	0.989	0.947	1.033
	PPD	-0.011	0.73	0.989	0.928	1.054
	PISA	0.000	0.43	1.000	0.999	1.002
	Constant	-4.520	0.01	0.011		
Step 7^a^	Gender	1.011	0.11	2.748	0.808	9.345
	BMI (≥ 25)	1.120	**0.02**	3.063	1.158	8.101
	Constant	-4.858	< 0.01	0.008		

BMI: body mass index, PPD: periodontal probing depth, PISA: periodontal inflamed surface area, significant values are highlighted in bold (p < 0.05)

## Discussion

*Summary of the main results*: The prevalence of periodontitis was high in the cohort. Parameters of periodontal inflammation, i.e. BOP and PISA were associated to more cardiac and systemic infections during observational period. Only age slightly correlated with survival, while a BMI of 25 or higher correlated with a three-fold increased risk of infections at heart/driveline.

*Comparison with published data*: To the authors’ knowledge, this is the first study, which evaluated the potential association of oral health parameters on the outcome in patients with HF, HTx and LVAD. Overall, the periodontitis prevalence and severity in this cohort was high. It is known that periodontitis is associated with heart failure; results from the population-based NHANES study in US showed that patients with moderate to severe periodontitis have a more than three times higher incidence of HF [18]. Another study found increased HF prevalence in patients with high concentrations of antibodies against potential periodontal pathogens [19]. Especially patients after HTx were found to suffer from severe periodontitis [20, 21], what is in line with the cohort in the current study. Considering the fact that different parts of the current study cohort were already investigated, showing a high periodontal burden [5, 6], those findings were expectable.

Main aim of this current study was therefore to reveal whether the oral situation of the patients would be associated with selected outcome parameters, including infections and survival. The current study revealed that periodontal inflammation, i.e. active/instable periodontal disease was associated with infections. It is documented that oral inflammation because of a polymicrobial biofilm increases the risk of bacteraemia, potentially leading to infections outside the oral cavity [22]. This bacteraemia can be caused by routine daily procedures and depends on (a) the quantity of bacteria and (b) the permeability of the junctional epithelium, which is both increased in case of periodontal inflammation [23–26]. Moreover, the local periodontal inflammation, microbiological burden and related endotoxins are also leading to a systemic inflammation, what is related to incident cardiovascular events [27, 28]. A hint for the relevance of this periodontitis-associated systemic inflammation has been found for HTx recipients, in which the serum high-sensitivity C-reactive protein was associated to periodontitis [29]. Accordingly, the relationship between periodontal inflammation and infections appears plausible. Further recent research supports the interaction between periodontal and vascular/cardiac tissues. It has been reported that, e.g., periodontitis and atherosclerotic diseases share similar inflammatory pathways [30]. In an animal model, periodontitis lead to a degeneration and hypotrophic changes of heart tissues [31]. Those two immunological and/or pathophysiological issues might underline an effect of periodontal infection as well as inflammation on cardiac changes and infections in the current study. Moreover, it has been shown that periodontitis would be associated with infectious complications at heart valves, especially in context of their surgical therapy [32, 33]. This supports the role of periodontitis in context of infectious complications. In a more generalistic approach, periodontitis affected the microbiome of different other tissues in the body, including liver, kidney and heart [34]. This might lead to the assumption that chronic periodontal inflammation could increase the infectious risk in individuals with severe heart diseases. While the current study found some singular associations, the effect of periodontal inflammation, especially on infections at heart/driveline was limited in the current study. In this respect, the literature regarding a potential effect of periodontitis on infectious complications of patients with severe HF seems plausible, but is not unequivocally substantiated by the current findings.

Interestingly, neither remaining teeth nor periodontal parameters were associated with survival in the current study. In contrast, literature shows that periodontitis severity as well as tooth loss can be confirmed as a risk predictor for mortality [3, 9, 10, 28]. Different potential explanations can be considered for this incongruence between current study and literature. First, the individuals within the current study cohort were very severely diseased or had undergone a serious therapy, i.e. HTx or LVAD. It can be seen that many deaths were caused by cardiological reasons, what might be irrespective from the periodontal status. Second, if one takes a closer look at the results (Tables 4 and 5) there were differences between different categories of remaining teeth and periodontal parameters, respectively, although they missed statistical significance. Especially recent studies evaluated the relationship between periodontitis and mortality in large samples over prolonged observational periods [3]. Therefore, the effect size might have been too small to reach significance in the current study sample. Third, within this retrospective study, patients underwent an oral examination between 2017 and 2018, whereby patients received a standardized letter to visit their family dentist [8]. This might have led to dental and/or periodontal therapy, what potentially affects the current findings. However, a previous prospective evaluation of a partial cohort one year after referral showed that the periodontal treatment need remained high over this period [8]. Thus, it can be assumed that maybe some patients would have undergone a respective therapy during the observational period, but the majority should have stayed periodontally diseased. However, periodontal inflammation is quite dynamic and can be modulated by nonsurgical therapy [35]. In contrast to the periodontitis severity (periodontal burden, stage), which does regularly not improve over time, the inflammation can be modulated easily. The presence or absence of inflammation at the time point of oral examination is thereby just a snap shot and could have differed during observational period. This makes an interpretation quite difficult.

None of the oral parameters was identified as a risk predictor for survival and cardiac infections in logistic regression models. In contrast, BMI was a strong predictor for cardiac infections. Increased BMI, combined with heart failure was found to be related to infections before [36]. It is known that obesity is affecting immunity, resulting in an increased risk of infections [37]. Therefore, the increased risk of patients with higher BMI to develop cardiac infections in the current study appears probable. Interestingly, periodontitis is related with obesity [38]. On the other hand, obesity can be protective against mortality in case of systemic infections (obesity paradox) [39]. Although no relationships in this respect can be revealed by the current study, this underlines the complexity of the patient cohort and thus the difficulties to draw robust conclusions on clinical consequences. Altogether, the current findings provide a hint for the need of improved periodontal care and maintenance in individuals with severe HF to support prevention of infections; however, a clinical evaluation of respective periodontal care concepts is still required.

Strengths and limitations: In this current study, a large cohort of patients with severe HF was included and comprehensively investigated. The consideration of dental and periodontal findings, including diagnosis following the recent classification of periodontal diseases alongside with gingival and periodontal inflammation strengthens the findings. On the other hand, the cardiological outcome parameters were various, including infections alongside with survival. The main limitation of the current study was its retrospective character. Thereby, it remained quite unclear, in which extend the oral situation of the participants has changed (positively in form of therapy or negatively in form of disease progression) during the observational period. Moreover, the cohort of severely diseased individuals had different factors, potentially influencing the results, e.g. co-morbidities, different forms of therapy etc. This makes the cohort quite heterogeneous and robust conclusions difficult. Furthermore, the majority of patients was periodontally diseased. Thereby, it is unclear what kind of effect can be expected when considering periodontitis as potential predictor. Regarding analysis of the potential relationship between survival and periodontal health, only overall survival was considered. Because the infection-related deaths were rare in the cohort, a distinct analysis of this point was not meaningful for this study, but should be considered for future research in the field. Altogether, more research in the field will be needed to understand the value of oral conditions, therapy and maintenance to support the systemic health of patients with severe HF.

## Conclusion

Patients with severe heart failure show a high periodontal burden, whereby periodontal inflammation might be associated with cardiac and systemic infections. Consequently, improved dental care, especially including periodontal therapy and maintenance appear required for patients with severe HF to support prevention of infections. Further large-scaled prospective studies are needed to understand the role of oral disease in cardiological outcome of individuals with HF and to prove a potential benefit or oral special care.

### Electronic supplementary material

Below is the link to the electronic supplementary material.

Supplementary Material 1

## Data Availability

The datasets generated and/or analysed during the current study are not publicly available due but are available from the corresponding author on reasonable request.

## Footnotes

1 PCP 15, Hu-Friedy, Chicago, IL, USA.

2 SPSS Inc., US.

## List of abbreviations

BMI: body-mass-index
BOP: bleeding on probing
CAL: clinical attachment loss
HF: heart failure
HTx: heart transplantation
LVAD: left ventricular assist device
PISA: periodontal inflamed surface area
PPD: periodontal probing depth
